# How to raise awareness about electronic mental health services among prospect healthcare providers: a qualitative study on information preferences

**DOI:** 10.1192/j.eurpsy.2022.745

**Published:** 2022-09-01

**Authors:** P. Braun, A.-K. Schwientek, L. Guthardt, A. Loerbroks, J. Apolinário-Hagen

**Affiliations:** Institute Occupational, Social and Environmental Medicine, Centre For Health And Society, Faculty Of Medicine, Düsseldorf, Germany

**Keywords:** semi-structured interviews, information preferences, medical and psychology students, eMental health

## Abstract

**Introduction:**

Since fall 2020, electronic mental health services (eMHSs) like apps can be prescribed by physicians and psychotherapists in Germany. However, future healthcare providers such as medical and psychology students remain reluctant to adopt eMHSs, even though they represent a vulnerable group with respect to developing mental health problems themselves. Reasons include scepticism and lacking awareness, which can be addressed by tailored multi-component information material. However, to date little is known about the most important information attributes to educate prospect healthcare providers on eMHSs.

**Objectives:**

The objective of this study is to explore information preferences on eMHSs among medical and psychology students.

**Methods:**

A total of 21 semi-structured interviews were conducted (n=16 medical and n=5 psychology students) across Germany based on a topic guide. Interviews were recorded, transcribed and content-analyzed using MAXQDA.

**Results:**

Most students reported having little knowledge about eMHSs and that the issue of digital health has never been raised in their study, even though it is perceived as important. Concerning information design preferences, students favored light, neutral colors and a combination of short, compressible texts with matching images. Regarding the content, information about data protection, the underlying evidence base and the match with personal needs were perceived as important for utilization intentions, while there was little interest in tailored information focusing exclusively on psychology or medical students.

**Conclusions:**

This study provides first insights into eMHS information preferences among prospect healthcare providers. In a next step, a discrete-choice conjoint experiment will be conducted to test the relevant information features on eMHSs.

**Disclosure:**

No significant relationships.

